# A Single Mass Forming Colonic Primary Mantle Cell Lymphoma

**DOI:** 10.1155/2016/2561507

**Published:** 2016-08-04

**Authors:** Fady Daniel, Hazem I. Assi, Walid Karaoui, Jean El Cheikh, Sami Bannoura, Samer Nassif

**Affiliations:** ^1^Division of Gastroenterology, Department of Internal Medicine, American University of Beirut Medical Center, P.O. Box 11-0236, Riad El Solh, Beirut 110-72020, Lebanon; ^2^Division of Hematology & Oncology, Department of Internal Medicine, American University of Beirut Medical Center, P.O. Box 11-0236, Riad El Solh, Beirut 110-72020, Lebanon; ^3^Pathology and Laboratory Medicine Department, American University of Beirut Medical Center, P.O. Box 11-0236, Riad El Solh, Beirut 110-72020, Lebanon

## Abstract

Mantle cell lymphoma (MCL) is a subtype of non-Hodgkin's lymphoma (NHL) comprising around 7% of adult NHL. It is characterized by a chromosomal translocation t(11:14) and overexpression of Cyclin D1. The incidence of secondary gastrointestinal tract involvement in MCL ranges from 10 to 28% in various series. However primary gastrointestinal MCL is very rare, accounting for only 1 to 4% of primary gastrointestinal lymphomas. The most common endoscopic feature of primary intestinal MCL is multiple lymphomatous polyposis. In rare cases it presents as protruded lesions or superficial lesions. Single colonic mass presentation is an extremely infrequent presentation. MCL has an aggressive course with quick progression, and most cases are discovered in the advanced stages. Colonic biopsies with histologic examination and specific immunohistochemical staining are the gold standard for a proper diagnosis. We report a case of a single mass forming mantle cell lymphoma of the ascending colon in a 57-year-old female patient with unusual colonoscopic and radiologic features and describe the therapy the patient received, thereby adding to the spectrum of clinical presentations of this aggressive lymphoproliferative disorder.

## 1. Introduction

Mantle cell lymphoma (MCL) is a small B-cell non-Hodgkin's lymphoma and is recognized as an aggressive B-cell lymphoma derived from a subset of naive pregerminal center cells with a propensity to involve extranodal sites. The gastrointestinal tract is the most common extranodal site involved by lymphoma, accounting for 5–20% of all cases of extranodal involvement [1]. Despite the high rate of secondary colonic involvement by MCL, primary gastrointestinal (GI) lymphomas are infrequently reported, accounting for only about 1–4% of all gastrointestinal malignancies [2]. The neoplastic cells are believed to originate from the mantle zone of the lymphoid follicles within the intestinal mucosa [3]. The molecular signature of MCL is an overexpression of the cyclin-D1 (CCND1) gene as a result of the chromosomal translocation t(11;14) that juxtaposes the protooncogene CCND1 to the immunoglobulin heavy-chain promoter [4]. As a result of this mutation, the lymphoma cells are usually immunohistochemically positive for cyclin-D1 and aberrantly coexpress CD5. Around 49% of MCL patients have gastroduodenal involvement, and specifically 38% to 62% have colorectal involvement [5, 6]. The difficulty in the diagnosis of primary GI lymphoma arises in part from the nonspecific and often benign gross endoscopic appearance. Of note, the most common endoscopic presentation of MCL is lymphomatous polyposis. In rare cases its presents as protruded lesions or superficial lesions [7]. MCL has an aggressive course with quick progression and most cases are discovered in the advanced stages. Primary MCL of the colon is a rare entity and its presentation as a single mass forming tumor is extremely unusual. We report a case of a single mass forming mantle cell lymphoma of the ascending colon in a 57-year-old female patient with unusual colonoscopic and radiologic features and describe the therapy the patient received.

## 2. Case Presentation

**Figure 1:**
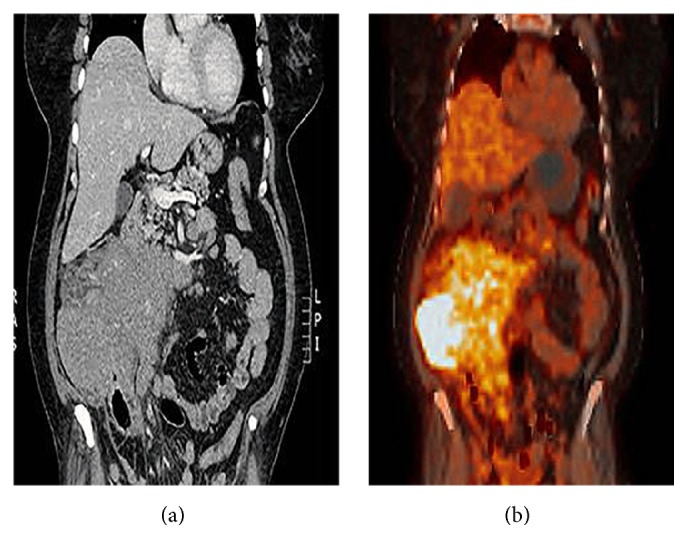
PET-CT scan image.

**Figure 2:**
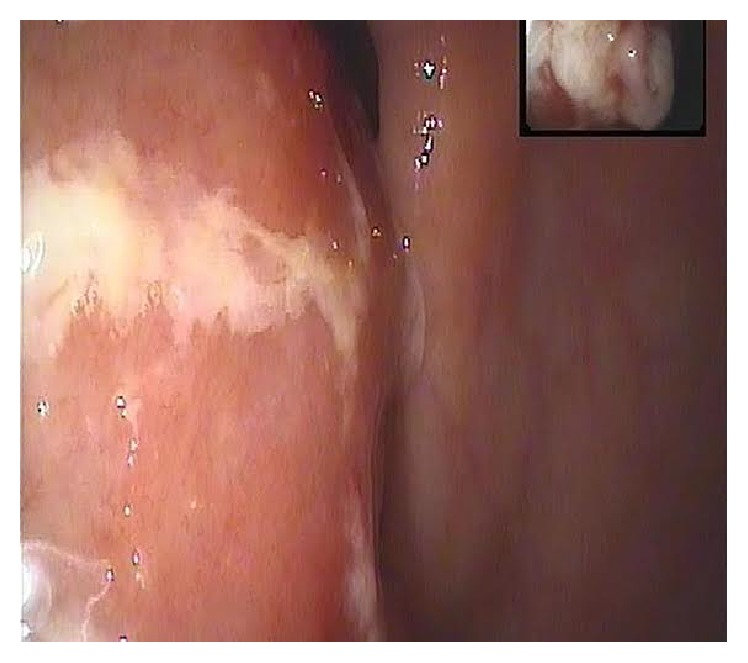
Endoscopic picture showing the colonic mass.

A 57-year-old woman with a history of hypertension and type 2 diabetes mellitus presented to our clinic with abdominal pain of 2 months duration, unintentional weight loss of 5 kg, night sweats, and fatigue. Abdominal physical examination revealed a palpable mass on the right side involving the right upper and lower quadrants at the level of the right colonic area. No organomegaly or palpable lymph nodes were noted. Her complete blood count was remarkable only for microcytic anemia, while chemistry studies including liver function tests and tumor markers (CEA, CA 19-9) were within normal limits. An abdominal CT scan showed a large soft tissue lesion arising from the wall of the ascending colon, with surrounding soft tissue deposits and enlarged pericolonic lymph nodes (Figure 1). Gastroscopy was normal with no evidence of gastritis or duodenal villous atrophy on corresponding biopsies. However, colonoscopy demonstrated a large fungating, ulcerated, and obstructing mass at the level of the ascending colon (Figure 2). The mass involved circumferentially the colonic wall with narrowing of the lumen, thereby preventing access to the terminal ileum. Additionally, the mass was friable and easily bleeding at scope contact. The remaining colonic segments were normal.

**Figure 3:**
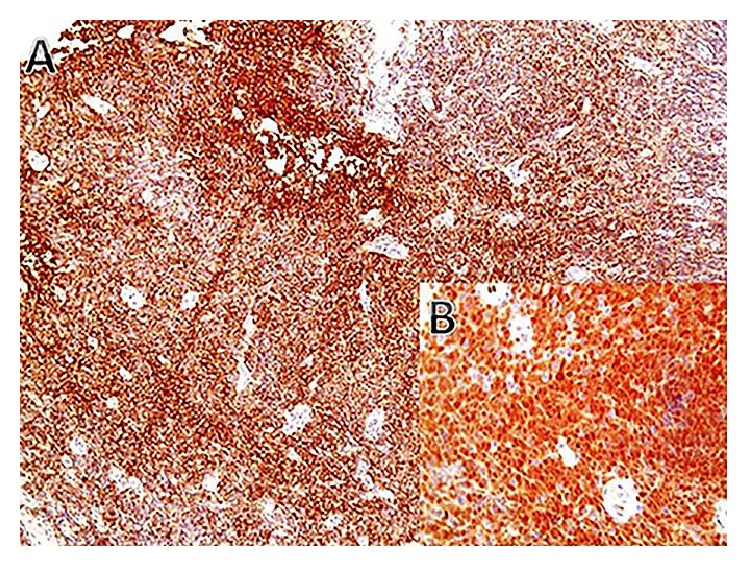
Immunohistochemical staining showing a diffuse CD20 positive B-cells ((A), 40x) coexpressing cyclin-D1 (B) (20x).

Biopsies were taken from the mass and showed lamina propria expansion by a diffuse population of medium-sized lymphocytes with mild to moderate nuclear irregularity and mildly increased mitotic activity (Figure 3). On immunohistochemical staining, the atypical lymphoid infiltrate was diffusely positive for CD20 and CD79s with coexpression of CD5, CD43, cyclin-D1, and BCL-2 and negative for CD3, CD10, CD23, and BCL-6. Proliferation index Ki-67 was around 40–50% (Figure 3). The morphologic and immunohistochemical findings were consistent with a mantle cell lymphoma.

Subsequently, staging radiologic studies were performed. Abdominal CT scan with intravenous contrast administration disclosed an enhancing solid mass replacing the entire right colonic area, with corresponding mesenteric blood vessels encasement. Positron Emission Tomography PET-CT scan showed hypermetabolic large right colonic mass with adjacent retroperitoneal, mesenteric lymph nodes, peritoneal deposits, and heterogeneous bone marrow uptake. Since no uptake was noted in the small bowel, balloon enteroscopy was not preformed. Additionally, a bone marrow core biopsy showed marrow involvement by MCL.

In view of the above findings, the patient's stage was determined as IV E as per the Ann Arbor classification. She was treated by induction chemotherapy alternating R-CHOP/R-DHAP for a total of 3 cycles of each protocol. A very good response was achieved, and the patient subsequently underwent conditioning with high dose chemotherapy (BEAM) followed by autologous stem cell transplantation (ASCT). Her overall treatment was well tolerated except for fatigue and grade 2 mucositis as well as febrile neutropenia. Evaluations after ASCT with a PET-CT scan on day 100 and day 180 showed a continuous complete remission. However, the patient refused colonoscopy after completion of chemotherapy.

## 3. Discussion

Mantle cell lymphoma is a subtype of non-Hodgkin's lymphoma (NHL) comprising around 7% of adult NHL [4]. It is characterized by a chromosomal translocation t(11:14) and overexpression of Cyclin D1 [4]. MCL commonly presents with advanced-stage disease, with about 80% of patients showing involvement of extranodal sites at presentation, including the bone marrow, spleen, Waldeyer's ring, and gastrointestinal tract [8].

Gastrointestinal tract involvement is recognized occasionally as being the presenting sign of lymphoproliferative disorders, and early recognition is important for staging, prognosis, and selection of appropriate treatment. Of note, the incidence of secondary gastrointestinal tract involvement in MCL ranges from 10 to 28% in various series [5]. At endoscopy, MCL in the intestines commonly manifests as numerous, small, spherical, or hemispherical polyps, a finding termed multiple lymphomatous polyposis (MLP) [2]. MLP can involve segments of the small intestine and large intestine, and the morphologic and immunohistochemical features of MCL presenting as MLP are similar to those of nodal MCL [9]. However primary gastrointestinal MCL is very rare accounting for only 1 to 4% of primary gastrointestinal lymphomas, and there is insufficient data to describe this rare entity [10]. The most common endoscopic feature of primary intestinal MCL is multiple lymphomatous polyposis [7]. Less commonly it presents as a single protruded lesion in the colon [7]. Ghimire et al. described seven cases of primary gastrointestinal lymphoma over a period of 11 years. Of these, three cases had colonic involvement with a diffuse morphology, one case showed gastric and colonic involvement, and one also showed rectal involvement [2]. In addition, Chung et al. described a series of seven cases of MCL over a period of 6 years. The majority of patients in their series were elderly and six out of seven cases had multiple polypoidal lesions ranging from 0.1 to 4 cm with central ulcerations. Additionally, diffuse polyposis was seen uniformly in their series, and polyposis was predominantly seen in the rectum and ascending colon, rather than in other sections of the colon [11]. Systemic chemotherapy usually consists of Cyclophosphamide, Adriamycin, Vincristine, and Prednisone plus Rituximab (R-CHOP) [5]. Dasappa et al. treated five patients with CHOP chemotherapy. Only one patient achieved complete remission and remained disease-free for 21 months before being lost to follow-up. The remaining four patients had inadequate response to CHOP chemotherapy with a median survival of 6 months [12]. Our patient was unusual in that she presented with a single large mass involving the right colon with luminal obstruction, with sparring of the remaining colonic segments. She received chemotherapy, followed by ASCT. Due to known poor survival in MCL and low response rate to systemic chemotherapy, we used an aggressive regimen alternating R-CHOP/R-DHAP followed by ASCT [13]. Seven months after transplant, the patient was still disease-free.

In summary, we described the case of primary colonic mantle cell lymphoma with an unusual presentation, thereby adding to the spectrum of clinical manifestations of this aggressive lymphoproliferative disorder. Although a rare entity, primary colonic lymphomas should be included in the differential diagnosis of single colonic lesions. Awareness of such occurrences is necessary and might help refine diagnostic methods for gastrointestinal lymphoproliferative disorders.
